# *In vivo* 3D Reconstruction of the Human Pallidothalamic and Nigrothalamic Pathways With Super-Resolution 7T MR Track Density Imaging and Fiber Tractography

**DOI:** 10.3389/fnana.2021.739576

**Published:** 2021-10-27

**Authors:** Dae-Hyuk Kwon, Sun Ha Paek, Young-Bo Kim, Haigun Lee, Zang-Hee Cho

**Affiliations:** ^1^Neuroscience Convergence Center, Green Manufacturing Research Center (GMRC), Korea University, Seoul, South Korea; ^2^Neurosurgery, Movement Disorder Center, Seoul National University College of Medicine, Advanced Institute of Convergence Technology (AICT), Seoul National University, Seoul, South Korea; ^3^Department of Neurosurgery, College of Medicine, Gachon University, Incheon, South Korea; ^4^Department of Materials Science and Engineering, Korea University, Seoul, South Korea

**Keywords:** pallidothalamic tract, H-field, fasciculus thalamicus, fasciculus lenticularis, ansa lenticularis, nigrothalamic tract, substantia nigra pars reticulata, thalamic substructure

## Abstract

The output network of the basal ganglia plays an important role in motor, associative, and limbic processing and is generally characterized by the pallidothalamic and nigrothalamic pathways. However, these connections in the human brain remain difficult to elucidate because of the resolution limit of current neuroimaging techniques. The present study aimed to investigate the mesoscopic nature of these connections between the thalamus, substantia nigra pars reticulata, and globus pallidus internal segment using 7 Tesla (7T) magnetic resonance imaging (MRI). In this study, track-density imaging (TDI) of the whole human brain was employed to overcome the limitations of observing the pallidothalamic and nigrothalamic tracts. Owing to the super-resolution of the TD images, the substructures of the SN, as well as the associated tracts, were identified. This study demonstrates that 7T MRI and MR tractography can be used to visualize anatomical details, as well as 3D reconstruction, of the output projections of the basal ganglia.

## Introduction

The basal ganglia (BG), which are broadly involved in various roles related to motor, associative, and limbic functions, are responsible for motor, cognitive, and mood changes typically caused by movement disorders such as Parkinson’s disease (PD; Obeso et al., 2014). Deep brain stimulation (DBS) for BG nuclei, such as the subthalamic nucleus (STN) and globus pallidus internal segment (GPi), has been accepted as an effective treatment for intractable and severe PD (Benabid et al., 2009). DBS surgical targeting relies heavily on an atlas consisting of a limited number of human postmortem specimens. In addition, while surgeons do their best to ensure that the electrodes are positioned within the STN in all patients through precise and detailed preoperative planning before surgery, there are several causes for the electrodes to deviate from the STN (Pinsker et al., 2008; Paek et al., 2013; Park et al., 2017). Therefore, three-dimensional (3D) visualization of the *in vivo* structure and connections of the human BG is important not only for understanding motor and neuropsychiatric diseases but also for supporting stereotaxic surgery that can minimize the side effects caused by DBS.

The final output of the BG is through the pallidothalamic and nigrothalamic tracts from the GPi and the substantia nigra pars reticulata (SNr) to the thalamus. However, despite the white matter (WM) structures around the SN, STN, and GPi being too complex, the schematic diagrams of these pathways are oversimplified compared to the actual connections (Hamani et al., 2017). It is difficult to apply observations of the pallidothalamic and nigrothalamic tracts in non-human primates to the human brain (Carpenter et al., 1976; Hazrati and Parent, 1991). In addition, even with the recently developed 9.4 Tesla (T) magnetic resonance imaging (MRI; Massey et al., 2012) and ultra-high-field 11.7T diffusion tensor imaging (DTI; Oishi et al., 2020), it is difficult to visualize in detail the entire course of the tracts *ex vivo*. Although the pallidothalamic pathway and related structures have been visualized with the surface models in a recent study using a sectioned cadaveric brain (Chung and Park, 2020), detailed reconstruction studies in 3D space are needed to determine the exact anatomy of the tracts *in vivo*.

Ultra-high field 7T MRI provides an opportunity to investigate the human brain at an enhanced signal-to-noise ratio (SNR), contrast, and resolution compared to low-field MRI (Cho et al., 2010). In addition, track-density imaging (TDI) has been developed as a means of creating super-resolution images of track density obtained from whole-brain tractography (Calamante et al., 2010, 2011, 2012). This technique generated a unique contrast, providing anatomical details as well as the high sensitivity of visualizing WM structures with a high contrast-to-noise ratio (Calamante et al., 2013; Cho, 2015; Cho et al., 2015a,b). The high TDI contrast combined with the improved sensitivity that can be achieved by 7T MRI suggests a possible role of the TDI for seed region definition and connectivity measurements through subsequent targeted fiber-tracking analysis of the human brain *in vivo*.

In this study, we obtained ultra-high-resolution structural and diffusion-weighted MR images of the human brain *in vivo* at 7T. We reconstructed the whole course of the pallidothalamic and nigrothalamic tracts and demonstrated the role of TDI in direct visualization of the subregions of the thalamus and SN in a narrow and complex region between the thalamus and the GPi and SN. The TDI maps and connectivity of the deep brain region obtained from this study will be helpful for a detailed understanding of the interaction between the BG and the thalamus, as well as for the study of mechanisms and surgical training for DBS.

## Materials and Methods

A 7T research MRI scanner (Siemens Magnetom) with an 8-channel radiofrequency head coil (Gachon University) was used for T2*-weighted images (T2*WIs) with 0.2 mm in-plane resolution and diffusion-weighted images (DWIs) with 1.8 isotropic resolution. DWI data along 64 directions with a b-value of 2,000 s/mm^2^ and *b* = 0 (b0) were obtained from a healthy 30-year-old adult male. MRI data were aligned with the anterior commissure (AC)–posterior commissure (PC) reference line. A T1-weighted whole-brain scan with 1-mm isotropic resolution was performed for anatomical reference using a 3T MRI scanner (Siemens Magnetom Verio). The details of the scan protocol are provided in (Supplementary Table 1). This study was approved by the Institutional Review Boards of the Gachon University of Medicine and the Korea Food and Drug Administration.

The DWI data were preprocessed to correct for geometric distortions (Oh et al., 2012). After the DWI data, including b0, were realigned and stacked, the fiber orientation distribution (FOD) in each voxel was calculated using a constrained spherical deconvolution (CSD)-based diffusion model with the MRtrix3 toolbox^1^. We then performed whole-brain tractography using the probabilistic iFOD1 algorithm with 35,000,000 streamlines, track minimum length = 20 mm, FOD amplitude threshold (cutoff) = 0.3, step size = 0.02 mm, and curvature radius constraint = 0.04 mm. The result was visualized by track-density imaging (TDI) implemented in the MRtrix3 with 0.2 mm isotropic resolution to match the T2*WIs (Calamante et al., 2010, 2011). The realigned and stacked T2*WIs were interpolated to have a 0.2-mm slice thickness and coregistered to the b0 volume image of DWI data using SPM12^2^.

The regions of interest (ROIs) were manually traced using MRtrix3 following the ROI-tracing framework for the hypointensity of iron-containing structures in T2*WIs (Kwon et al., 2012). The ROIs were determined as the regions that maintained shape and contrast continuously through all slices in T2*WIs. After the widest portion of the ROIs was defined, the remaining ROIs were delineated as they became progressively visible in the horizontal slices. The boundary of the next adjacent slice was defined based on the previous slice to avoid any sudden changes in shape. The same process was repeated in the coronal and sagittal slices. The proximity and similar signals in T_2_*WIs can make it difficult to discriminate the structures around the ROI. Whole-brain TDI images, which can provide excellent contrast of the white matter areas (Cho, 2015), were introduced to confirm the boundary delineated by the T2*WIs. However, because the TDI images could not provide anatomical details, the TDI images were used only to correct or confirm the boundary. Using these tracing methods, the GPe, GPi, STN, and SN were identified (Figure 2). The thalamus was segmented on T1-weighted images (T1WIs). T1WIs were registered through linear rigid-affine transformation to the b0 volume image of DWI scans. The 3D Slicer^3^ was used for 3D modeling.

**Figure 1:**
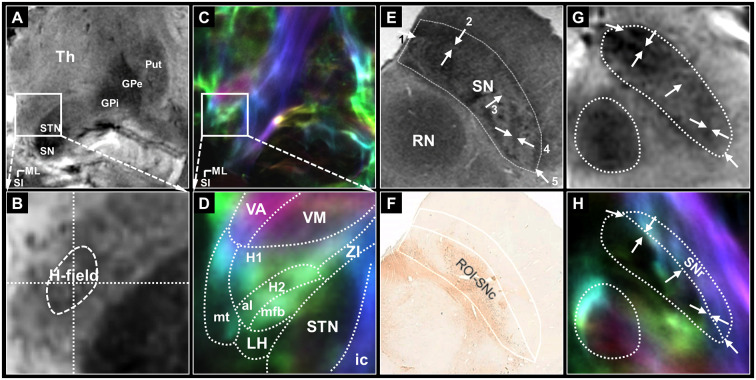
The seed regions for tractography of the pallidothalamic and nigrothalamic pathways. **(A,B)** The selected (top) and magnified (bottom) views of the seed regions, H-field, obtained with 7T T2* MR imaging. **(A)** An image obtained with ultra-high-field MR imaging clearly shows the putamen, GPe, GPi, STN, and SN. **(B)** In the middle of the image, H-field is observed as a darker region containing a hypointense signal in the T2* MR image than the surrounding WM regions. **(C,D)** The corresponding views were obtained with super-resolution DEC-TDI maps showing the representation of directionality and density. Enlarged view of the Fields of Forel with anatomy depicted by white dotted lines drawn on a coronal section 33 (14.6 mm posterior to the AC level) modified from the stereotactic atlas of Mai (Mai et al., 2008) **(D)**. **(E,F)** In *ex vivo* imaging, the whole SN ROI corresponding to most of SN hypointensity on the 7T T2* MR image **(E)**, the SNc ROI based on the TH stain within the whole SN including the A9 cell group **(F)** adapted from Lee et al. (2016). **(G,H)** The corresponding section of the SN from the T2* **(G)** and TDI **(H)** images in this study. White arrows, such as those shown in **(E)**, indicate the referenced anatomical landmarks for parcellation of the SNr. The SNr ROI is based on the TDI mapping within the whole SN **(H)**. For anatomical annotations and orientation abbreviations, see those described in Figure 2 captions. H1, fasciculus thalamicus; H2, fasciculus lenticularis; LH, lateral hypothalamic area; ZI, zona incerta.

**Figure 2:**
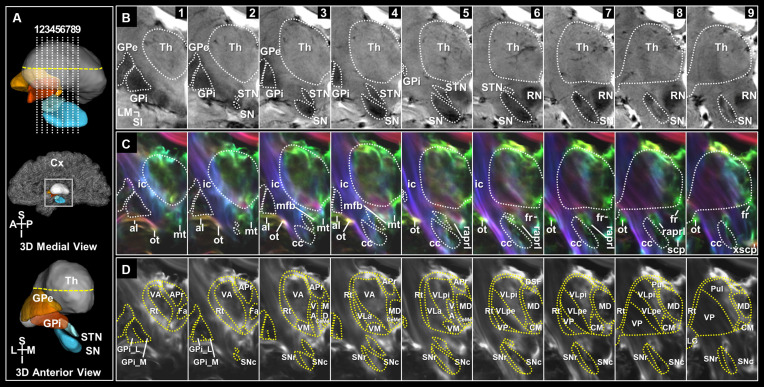
Coronal sections of the deep brain regions and surrounding WM tracts in the super-resolution TDI maps. **(A)** Slice levels shown in a 3D representation of the deep brain regions with imaging locations shown from a midsagittal view (top). 3D model of those shown from an anterior view (bottom). **(B)** The coronal view images of the deep brain on T2*-weighted MRI at 7T in serial 2 mm sections from anterior to posterior levels. **(C)** Super-resolution DEC-TDI maps of the deep brain regions and surrounding WM structures. Fiber-directionality information was incorporated as red (right-left)–green (anterior-posterior)–blue (superior-inferior). **(D)** Super-resolution TDI maps tracing the substructures of the thalamus and BG in selected slices. Anatomical annotations based on the stereotactic atlas by Mai (Mai et al., 2008). al, ansa lenticularis; APr, anteroprincipal nucleus; cc, crus cerebri; CeMe, central medial nucleus; CM, centromedian nucleus; Cx, Cortex; DSF, dorsal superficial nucleus; Fa, fasciculosus nucleus; fr, fasciculus retroflexus; GPe, globus pallidus, external segment; GPi, globus pallidus, internal segment; GPi_L, GPi lateral part; GPi_M, GPi medial part; ic, internal capsule; LG, lateral geniculate nucleus; MD, mediodorsal nucleus; mfb, medial forebrain bundle; mt, mammillothalamic tract; ot, optic tract; Pul, pulvinar; raprl, prelemniscal radiation; RN, red nucleus; Rt, reticular nucleus; scp, superior cerebellar peduncle; SN, substantia nigra; SNc, SN pars compacta; SNr, SN pars reticulata; STN, subthalamic nucleus; Th, thalamus; VA, ventral anterior nucleus; VLa, ventral lateral anterior nucleus; VLpe, ventral lateral posterior nucleus, external part; VLpi, ventral lateral posterior nucleus, internal part; VM, ventral medial nucleus; VP, ventral posterior nucleus; xscp, decussation of scp. Orientation: A, anterior; I, inferior; L, lateral; M, medial; P, posterior; S, superior.

Seed regions were defined to identify the pallidothalamic pathways. The H-field was selected for tracing the entire pallidothalamic tracks in the coronal section of the Fields of Forel, as shown in Figures 1A–D. The H-field is a candidate for an excellent seed region for tracing related connections as a pathway into the thalamus through the fasciculus thalamicus (H1) after the convergence of two major streams of the pallidothalamic pathway (Gallay et al., 2008), the ansa lenticularis (al) and fasciculus lenticularis (H2). Since WM contrast is dependent on fiber orientation in gradient echo MRI (Wharton and Bowtell, 2012), just as the H2 bundle typically shows hypointensity in the coronal T2* MR image, the H-field, which shows directionality and high density in the TDI map, indicates a hypointense signal lower than the signal intensity of surrounding Fields of Forel (Figure 1B). The TDI contrast can therefore play an important role in accurately defining the H-field by showing the anatomy of the major WM distribution in the complex subthalamic area that T2* MR images cannot show in detail (Figure 1D). The H-field defined in the T2* MR image includes the boundary between H1 and H2 and the area around it.

The SN pars reticulata (SNr) regions were also selected for tracing the entire nigrothalamic tracks in the coronal sections of the SN, as shown in Figures 2E–H. Both SN pars compacta (SNc) and SNr of the SN are tissues that contain brain iron and show similar image contrast in T2* MR images. However, it is possible to divide the SNr from the SNc in parallel with tissue staining in the post-mortem MRI study (Lee et al., 2016). To distinguish SNr from SNc, we were able to find several characteristic landmarks similar to and consistent with our T2* image contrast from the results of Lee et al. (2016): (1) the outer boundary of the narrow hyperintensity region between the hypointensity regions on the ventromedial side (Figure 1E arrows 1 and 2; (2) the outer boundary of the brighter area (dorsal side) from the contrast difference between the ventromedial and dorsolateral areas (Figure 1E arrow 3; and (3) the inner border of the brighter area (ventrolateral side) from the contrast reversal of the dorsolateral area (Figure 1E arrows 4 and 5). As a strong candidate for a structure that induces these distinct contrast differences in 7T T2* images of the SN is the nigrosome belonging to the SNc (Damier et al., 1999), the pocket-like regions, which are hyperintensity than their periphery, partially overlap with the location and shape of the nigrosomes (Kwon et al., 2012). The rostral level with relatively weak overlap with the nigrosome structure indicated a difference in contrast between the two subregions of the SN in the DEC-TDI image (Supplementary Figure 1B). At the intermediate level of the SN, the above-mentioned landmarks were followed (Figure 1G and Supplementary Figure 1C), and at the caudal level, the SNr were parcellated based on the boundary between the internal hyperintensity structure and the outer hypointensity region (Supplementary Figure 1D). Following the manual ROI-tracing method above, the SNr seed regions were finally defined in the entire SN volume (Figures 1H, 2D, and Supplementary Figures 1B–D).

Probabilistic CSD-based fiber tracks were generated from the seed regions using the iFOD2 algorithm with the default tracking parameters except for the cutoff (0.4) and the number of selected streamlines (pallidothalamic pathway: 35,000,000, nigrothalamic pathway: 6,000,000; Tournier et al., 2010). The initial tracks were filtered by identifying tracks connected with GPi, SNr, and thalamus masks and then extracted by maximum length (pallidothalamic pathway: 50 mm, nigrothalamic pathway: 40 mm) and by excluding ROIs (pulvinar, pallidothalamic pathway: red nucleus, nigrothalamic pathway: GPi and periaqueductal gray).

## Results

**Figure 3:**
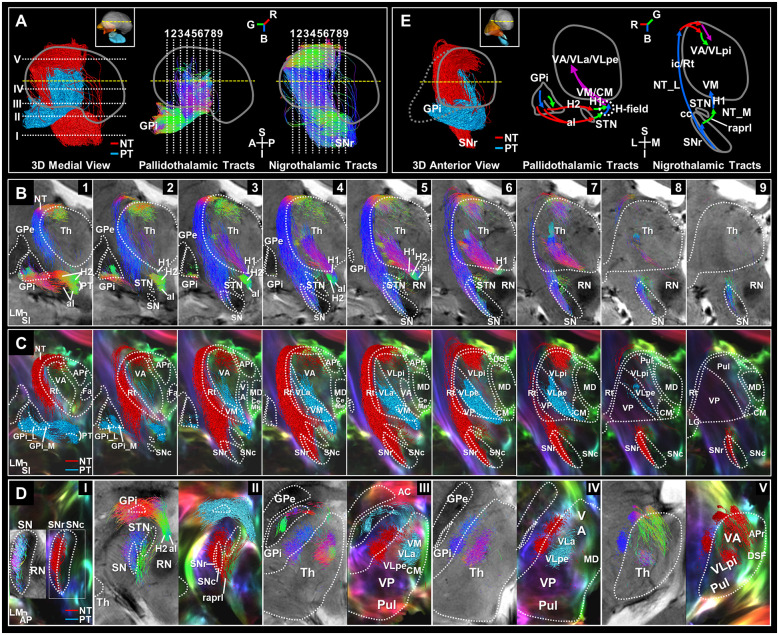
Separation of the basal ganglia output pathways. **(A)** 3D color-labeled and directional color-represented streamline tracks in midsagittal view. Slice levels are shown in 3D streamlines of the pallidothalamic (PT) and nigrothalamic (NT) pathways corresponding to the slice numbers atop **(B)** and **(D)**. Directional color represents track directionality [R, red (ML), G, green (AP), B, blue (SI)]. **(B)** 7T T2*-weighted images overlaid with the directional color-encoded tracks of the PT and NT pathways in serial coronal views with 2 mm spacing from anterior to posterior, aligned to the slice levels in Figure 2. **(C)** Super-resolution DEC-TDI maps overlaid with the 2 mm thick reconstructed color-labeled pathways (PT: red label; NT: light blue label). **(D)** Streamline tracks showing directionality (left column) and category (right column) were reconstructed to 1 mm thickness and superimposed in each axial view of the T2* and DEC-TDI images. **(E)** 3D color-labeled streamline tracks in anterior view (left). Overall, coronal schematic diagrams of the PT (center) and NT (right) pathways are shown. The path-directionality information is incorporated as an RGB color representation. Anatomical annotations and orientation abbreviation are the same as described in Figure 2 captions. AC, anterior commissure; H, H-field; H1, fasciculus thalamicus; H2, fasciculus lenticularis; NT_L, NT lateral pathway; NT_M, NT medial pathway.

We identified the entire path of the pallidothalamic and nigrothalamic tracts and substructures of the thalamus, GPi, and SN in super-resolution T2* and TDI images with an in-plane resolution of 0.2 mm (Figure 2 and Supplementary Figure 2). Based on the sectional image and the streamline obtained from tractography, it was arranged with a reconstructed 3D model to provide a comprehensive understanding of the structures *in vivo*. In three imaging planes, we visualized the entire pathway from the BG to the thalamus in detail. Each pathway was encoded with directional colors (red: right-left, green: anterior-posterior, blue: superior-inferior) to indicate spatial directionality (Figures 3B,D) and labeled with different colors (pallidothalamic pathway: red label, nigrothalamic pathway: light blue label) to show the distribution within the thalamus (Figures 3C,D and Supplementary Figure 3).

### Substructure of the Basal Ganglia and Thalamus

Figure 2 shows images of selected brain areas, including the BG and thalamus. Figure 2A shows representative 3D models of the deep brain regions reconstructed using submillimeter voxels. As shown in Figure 2B, 7T T2* MR images clearly showed the putamen, GPe, GPi, STN, and SN. Moreover, the internal structures that overlapped with the location of the nigrosomes at the intermediate and caudal levels of the SN were also clearly visualized (Figures 2B, 7–9; Supplementary Figure 1, and Supplementary Figure 2—p1-p2). This result is consistent with our previous report (Kwon et al., 2012). Substructures of the GPi, SN, and thalamus are identifiable in super-resolution TDI images. Furthermore, by incorporating directionality information, the super-resolution directionally encoded color TDI (DEC-TDI) maps enhance the boundaries between the various substructures (Figure 2C and Supplementary Figures 2, 3). As shown in Figure 2D, we were able to identify substructures, such as the GPi medial (GPi_M) and GPi lateral (GPi_L) parts, substantia nigra pars compacta (SNc) and reticulata (SNr), centromedian nucleus (CM), ventral anterior (VA), medial (VM), and posterior (VP) nuclei, and ventral lateral (VL) anterior (VLa) and posterior (VLp) nuclei, among others. To interpret the extent of the labeling, more sections were displayed in three planes containing the level of the AC anteriorly and the level of the pulvinar posteriorly (Figure 3D and Supplementary Figures 2, 3).

### Pallidothalamic Pathway

Figure 3B shows direct visualization of the al and H2 of the pallidothalamic pathway. The al mainly originates from the inferior side of the GPi_L and some from the superior side (Figure 3B—1–3). The H2 appears to originate from the whole extent of the GPi and is mainly emitted *via* the GPi_M (Figure 3B—1–3). The direction of the pallidothalamic tracts starting from the GPi goes to the medial side and is encoded in red color when crossing the internal capsule, where al is mainly distributed on the inferior side, while H2 is distributed on the superior side (Figure 3B—I, Figure 3D—II and Supplementary Figures 3—6), but the direction immediately detours in front of the STN and proceeds to the posterior side (Figure 3B—1–3 and Figure 3D—II), which is encoded in green color. H2 maintains its direction until it joins H1, but al proceeds from a slightly inferior portion than H2 and is merged into the H-field in the superior portion, so it is encoded in blue while heading to the superior side within the H-field. The al is mainly distributed on the medial side within the H-field, while H2 is distributed on the lateral side in directional color images (Figure 3B—4, 5 and Figure 3D—II). As the pallidothalamic pathway enters the thalamus through H1 running from its ventromedial to dorsolateral side, it changes its direction as encoded in purple color (Figure 3B—2–6). Finally, the pallidothalamic projections show ventral distribution traveling to the CM, VM, VA, VLa, and VLp external part (VLpe) through the track bundle originating from H1 (Figure 3C, Figure 3D—III–V, and Supplementary Figure 3B).

### Nigrothalamic Pathway

The nigrothalamic pathway is characterized by two pathways. In the lateral path, nigrothalamic tracts originating from the SNr (Figure 3B—3–9, Figure 3D—I, II, and Supplementary Figure 3B—3–5) proceed along the surface of the thalamus through the internal capsule and reticular nucleus (Rt) to the superior portion of the anterior thalamus, then penetrate toward the dorsal part of the VA and VLp internal part (VLpi), and in the medial path, some fiber tracks originating from the caudal SNr send a tributary to the VM. The medial path of the nigrothalamic pathway consists of tracks where some tributaries exit in the medial direction from caudal SNr through SNc and proceed to the anterior side direction through the prelemniscal radiation (raprl; Figures 3B,C—5–9, Figure 3D—II, and Supplementary Figure 3B—3, 4), and then changes direction near the VM and enters through the upward H1 encoded by blue color (Figure 3B—3, 4, Figure 3D—III, and Supplementary Figure 3B—5). The direction of the lateral path of the nigrothalamic tracts originating from the SNr is expressed as blue streamlines ascending along the vertical direction of Rt and the internal capsule of the lateral side of the thalamus (Figure 3B—1–7 and Figure 3D—III–V) to enter the superior portion of the VA and VLpi for tracks (Figure 3C—1–8, Figure 3D—V, and Supplementary Figure 3B—2–5). Then, it changes to the medial direction (red color, Figure 3B—1–8, and Figure 3D—V) toward the VA and VLpi that surround the inside of the thalamus. Finally, it is composed of green-colored tracks penetrating toward the VA to the posterior side and purple-colored tracks progressing directly to the ventromedial direction from the dorsolateral direction to enter the VLpi (Figure 3B—1–7 and Figure 3D—V).

Overall, the schematic diagrams represent the entire directionality of the pathways (Figure 3E). The thalamic projections from the BG could be summarized into two patterns, “>”-type connections *via* pallidothalamic pathways (Figure 3E center) between GPi and predominantly VM, CM, VA, and VL and “F”-type connections *via* the nigrothalamic pathways (Figure 3E right) between SNr and predominantly Rt, VA, VL, and VM. The whole course of these BG output pathways is summarized in Table 1, giving details of the identified connections in different thalamic nuclei.

**Table 1:** Summary of the pallidothalamic and nigrothalamic pathways.

Tracts	Starting point	Course (anatomical landmarks)	Endpoint
Pallidothalamic pathway	Whole GPi	Anterior GPi_M → H2 (higher level when crossing ic) → Between anterior STN and rostral ZI → H-field (lateral side) → H1	VM, CM, Ventral VA, VLa, VLpe
	Superior and inferior GPi_L	Anterior GPi_L → al (lower level when crossing ic) → Between anterior STN and rostral ZI → H-field (medial side) → H1	
Nigrothalamic pathway	SNr	cc → ic and Rt	Dorsal VA, VLpi
		raprl → H1	VM

*Anatomical annotations are the same as described in Figure 1 captions. ZI = zona incerta*.

## Discussion

We performed tractography of an *in vivo* human deep brain and generated detailed reconstructions of the whole course of thalamic connections with BG nuclei, such as the GPi and SNr, using ultra-high field 7T diffusion MRI. In particular, the nigrothalamic pathway, which has received less attention than the pallidothalamic pathway and did not show detailed pathways (Lenglet et al., 2012), was visualized in three dimensions. To achieve this, we used a careful and conservative approach for the analysis and interpretation of the data. Accordingly, we took the following methodological precautions: (1) to ensure high accuracy in estimating the principal direction of the diffusion tensor, the images were acquired using a protocol optimized based on high angular resolution diffusion imaging (64 directions, b-value = 2,000) with a 7T MRI with high SNR (Tuch et al., 2002; Tournier et al., 2004); (2) misregistration arising from geometric distortions of the original images acquired using the echo-planar technique were corrected; (3) the seed regions were defined using high-resolution 7T T2* MR images and TDI with a 0.2 mm in-plane resolution, which is smaller than the fiber orientation distribution (FOD) resolution of 1.8 mm^3^; (4) we chose to use the tractography algorithm, iFOD2, which is capable of tracking with high accuracy through both highly curved and crossing fiber regions; and (5) and to examine projection characteristics along major tracks within the thalamus, remote from but structurally connected to the GPi and SNr regions, we located the seed regions directly from the super-resolution TDI image. This approach allowed us to position the seed image more accurately in fiber tracking (Calamante et al., 2013). As a result, we were able to identify the seed regions of the H-field more accurately for tracing the pallidothalamic pathway and SNr for tracing the nigrothalamic pathway.

As the H-field can be used directly in the tracing of the pallidothalamic pathway, several problems that may occur when performing tractography by setting GPi as the seed region can be solved: When GPi is set as the seed region, it is highly likely that more false-positive results than tractography results in one section of the H-field can be included. Setting the H-field as a seed region can therefore reduce the constraints and subjectivity for extracting the pallidothalamic pathway. Accordingly, using the H-field was able to solve the problem that the connection strength of the pallidothalamic pathway through the H-field may be relatively decreased among the connections between the GPi and thalamus.

Unlike the pallidothalamic pathway, which includes the anatomically clear and well-known WM pathways in Fields of Forel, defining the SNr in track-tracing is important because the nigrothalamic pathway has no well-known anatomical landmarks that can be selected as a seed region in the nigrothalamic pathway. The 7T T2* imaging allows us to identify the internal structures that overlap with the location of the nigrosomes at the intermediate and caudal levels of the SN because of the high paramagnetic contrast of iron-containing structures (Kwon et al., 2012; Kim et al., 2016). However, the proximity and similar signals between the SNr and the SNc in T2*-weighted MR images make it difficult to discriminate between the two structures *in vivo*. In addition, the low resolution of diffusion MRI also made it difficult to define SNr as the seed region (Tan et al., 2015). These factors make visualization of the nigrothalamic pathway difficult. However, in this study, the entire course of the nigrothalamic pathway could be traced more accurately by defining the SNr as the seed region with an isotropic resolution of 0.2 mm.

To examine the BG projection patterns of each functionally different thalamic sub-region, the TDI images creating 200 μm super-resolution images of track density originating from whole-brain tractography were introduced in this study. TDI technique generated the unique contrast of providing anatomical details as well as the high-sensitivity of WM structures visualization with a high-contrast-to-noise ratio (Calamante et al., 2013; Cho, 2015; Cho et al., 2015a,b). The contrast in the TDI maps revealed gray matter (GM) or compact structures caused by fiber track density near the border of the substructures surrounded by the WM or reticular structures. As the GM is a representative isotropic diffusion area in diffusion-weighted imaging, we found that the SNc structures could be shown as a region of signal hypointensity in the TDI maps, on the other hand, the SNr or Rt with a reticular structure show hyperintensity with directionality (Figures 1, 2 and Supplementary Figures 1–3). The TDI contrast is thus helpful for the separation between substructures in the deep brain nuclei. In this study, the TDI showed many thalamic and nigral substructures which correlated well with known anatomy from a histological human brain atlas (Mai et al., 2008), including the complex WM tracts surrounding these nuclei (Figures 2, 3 and Supplementary Figures 2, 3). In addition, a recent study demonstrated the histologically guided parcellation of thalamic nuclei based on short-track TDI (stTDI) with very high reliability, using 3T MRI scans from the Human Connectome Project database (Basile et al., 2021).

After taking these careful steps, our tractography results show that thalamic connections with BG nuclei are associated with the representative motor thalamus, VA, VM, VLa, and VLp, in terms of spatial connectivity. More closely, afferents from SNr are found mainly in VA and VM nuclei, afferents from GPi preferentially target the VA and VLa nucleus (Bosch-Bouju et al., 2013). Although it is unknown whether all tracks identified by data-driven tractography represent true axonal fibers, whole-track streamlines can yield false-positive results by including numerous passing tracks. On the other hand, track end-points, which are more likely to show the fiber connecting zone, may have false-negative results by missing the connecting zone through the branches of the passing fibers. These whole-track modalities, including track end-points, must thus be considered. In addition, it should be noted that diffusion-based tractography cannot infer whether a fiber projects onto a structure or passes through it. It might, therefore, be that some of these reconstructed tracks from the GPi merely traverse through the VM without projecting onto them.

Interestingly, although some thalamic regions receive connections from both GPi and SNr, these two afferents might not overlap, except for VM. In particular, the fact that the VA pathways are connected in a separate space suggests that they play the same role of sending the final GABAergic signals of the BG to the thalamus, but that there is a difference in function depending on the location where they are connected. This is in line with recent reports describing various functions of the VA (Gorka et al., 2020). The BG, which has been regarded to control movement through the simple inhibition of the thalamus, is expected to be involved in more detailed regulation through two connection channels with different pathways and different thalamic targets. That is, even if the BG output is shared by the GPi and the SNr, if the path and thalamic target overlap, there is no need to divide it into two output channels. Differences in the anatomical locations of the GPi and SNr create independent pallidothalamic and nigrothalamic connections. It can be speculated that this may eventually lead to differentiation of BG output functions for the thalamus, as it allows the GPi and SNr to connect with different thalamic target nuclei.

These two projections of the GPi and SNr and their inputs to the thalamus are similar but different in animal data. Similar to the pallidothalamic pathway observed in humans *in vivo*, unilateral injections of the anterograde tracer in the GPi of the non-human primate led to anterograde labeling of fibers ipsilaterally in the VA, VL, CM, and lateral habenula. The labeled fibers traveled along or through the al, H2, H1, and the Forel’s fields (Hazrati and Parent, 1991). However, contrary to the nigrothalamic pathway observed in humans *in vivo*, the nigrothalamic fibers project through the H-field and then produce two branches. The medial component gives rise to an anterior branch and projects medially to the VA and MD. The lateral component produces a posterior branch, which proceeds laterally to the posterior part of the VA, and the subdivisions of the MD (Francois et al., 2002; Neudorfer and Maarouf, 2018). On the other hand, a study in the rat reported that a small injection of the anterograde tracer into the SNr revealed two populations of SNr neurons projecting onto the VL and VM (Kha et al., 2001).

The functional difference between these two pathways may provide a further understanding that can explain the mechanistic differences between GPi- or STN-DBS, which are implemented to ameliorate the symptoms of advanced Parkinson’s disease. Although the mechanism of action of DBS is still not fully understood, in principle, DBS can directly activate the target nuclei and nearby areas and would bring about orthodromic effects in the downstream direction (Li et al., 2014). Therefore, it can be speculated that DBS in the STN compared to GPi-DBS not only simultaneously stimulates two pathways of functionally different BG output *via* the GPi and the SNr, but also potentially produces a wider range of effects by stimulating thalamic nuclei located at different locations in the thalamus site.

Our study has some limitations. First, this is a single subject work, and the contralateral pathways were not included. However, the advantages of high-resolution imaging studies are that they can allow for direct identification of small structures of the human brain *in vivo*, as well as individual observations rather than averaging over multiple subjects. The super-resolution TDI images show some degrees of similarity to anatomical boundaries because additional anatomical information derived from signal modeling and tracking algorithms are incorporated. However, tractography-based methods for parcellation of thalamic and nigral structures are influenced by the well-known limitations related to streamline density (Jones et al., 2013). In addition, except for noise increase in tractography by fiber tracking on lower spatial and angular resolution, the nature of the fiber track distributed across tissues can blur the anatomical boundaries in TDI images, and this effect may cause a difference in parcellation between raters. However, these limitations can be compensated using a multimodal imaging method such as combining structural 7T MRI, which exhibits high inter-rater reliability (Kwon et al., 2012).

In conclusion, we defined the H-field as a seed region for tracing the pallidothalamic pathway and the SNr for the nigrothalamic pathway using super-resolution 7T T2* MRI and TDI. As a result, we visualized 3-dimensionally two different connections of the BG output to the thalamus in the human brain *in vivo* and generated a comprehensive map of these pathways with distinct projection patterns into different thalamic nuclei. Thus, we believe that the TDI technique is helpful for the separation between substructures in the deep brain regions, and our findings will be critical for further understanding and surgical training for GPi- and STN-DBS.

## Data Availability Statement

The original contributions presented in the study are included in the article/Supplementary Materials, further inquiries can be directed to the corresponding author/s.

## Ethics Statement

The studies involving human participants were reviewed and approved by Institutional Review Boards of the Gachon University of Medicine and Korea Food and Drug Administration. The patients/participants provided their written informed consent to participate in this study.

## Author Contributions

D-HK designed the study, performed data processing, and wrote the draft of the manuscript. Z-HC formulated the super-resolution tractography and its application to neural circuitry. SP and Y-BK participated in neurological and surgical discussions. HL supported overall research. All authors contributed to the article and approved the submitted version.

## Conflict of Interest

The authors declare that the research was conducted in the absence of any commercial or financial relationships that could be construed as a potential conflict of interest.

## Publisher’s Note

All claims expressed in this article are solely those of the authors and do not necessarily represent those of their affiliated organizations, or those of the publisher, the editors and the reviewers. Any product that may be evaluated in this article, or claim that may be made by its manufacturer, is not guaranteed or endorsed by the publisher.

## Footnotes

^1^http://www.mrtrix.org; Tournier et al., 2012

^2^ https://www.fil.ion.ucl.ac.uk

^3^ http://www.slicer.org
