# Supplementary material for: In vivo 3D Reconstruction of the Human Pallidothalamic and Nigrothalamic Pathways With Super-Resolution 7T MR Track Density Imaging and Fiber Tractography
Source: Front Neuroanat. 2021 Oct 27;15:739576. doi: 10.3389/fnana.2021.739576 (PMC8579044; doi:10.3389/fnana.2021.739576)
Supplement: Supplementary file 1 [file Data_Sheet_1.docx]

Supplementary Material

# Supplementary Table

**Supplementary Table 1.** Scan parameters for the whole-brain 7T MRI, images [1]–[4], and 3T MRI, image [5].

Image [1] parameters were applied to 2-dimensional planning sequence before Diffusion-weighted (DW) echo planar imaging (EPI). To obtain the reference line, a single-slice T1-weighted magnetization-prepared rapid acquisition with gradient echo (MPRAGE) sagittal image was acquired in the midline. Image [2] parameters were those of DW EPI. Fast imaging techniques, such as GRAPPA (reduction factor = 3) were used to reduce scan times. Parameters of image [3] and [4] were used for MR images. Image [5] parameters were applied to 3T T1-weighted MR imaging for the anatomical information. TR = repetition time; TE = echo time; FA = flip angle; BW = bandwidth; Th = slice thickness; NEX = number of excitation; GRE = gradient echo; GR = gradient recalled echo; IR = inversion recovery; GRAPPA = generalized autocalibrating partially parallel acquisition.

| Image | Plane | Sequence | TR  (ms) | TE  (ms) | FA  (degree) | BW  (Hz/pixel) | Matrix | Slice | Th  (mm) | NEX |
| --- | --- | --- | --- | --- | --- | --- | --- | --- | --- | --- |
| [1] T1 | sagittal | MPRAGE | 4000 | 2.77 | 10 | 470 | 256 × 256 | 1 | 4 | 32 |
| [2] DW | axial | EPI | 6000 | 83 | 90 | 1562 | 128 × 128 | 45 | 1.8 | 3 |
| [3] T2* | coronal | GRE | 750 | 21.6 | 30 | 30 | 1024 × 864 | 88 | 2 | 1 |
| [4] T2* | axial | GRE | 750 | 21.6 | 30 | 30 | 1024 × 864 | 80 | 2 | 1 |
| [5] T1 | 3D | GR/IR | 1900 | 2.93 | 9 | 170 | 224 × 256 | 176 | 1 | 1 |

# Supplementary Figures


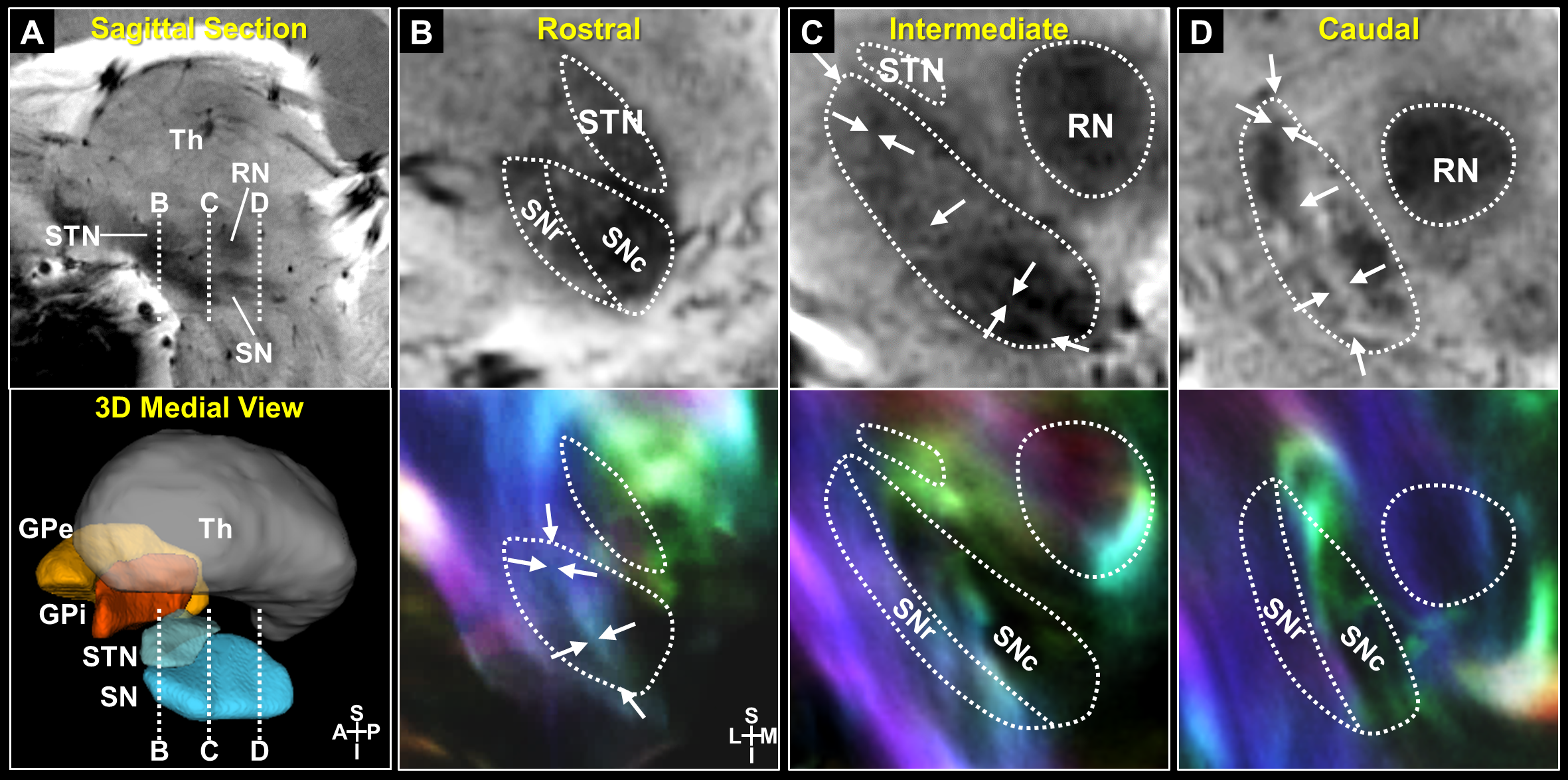


**Supplementary Figure 1.** Coronal sections of the SN subdivisions illustrated at three representative levels.

(A) White dotted lines are parallel to the coronal plane at the levels corresponding to the slices shown in (B, C, and D). 7T T2* (top) and corresponding DEC-TDI (bottom) images show the rostral (B), intermediate (C), and caudal (D) midbrain. These selected sections correspond to slice numbers 3, 6, and 9 in Fig. 2B and C. White arrows indicate the referenced anatomical landmarks for parcellation of the SNr: in (B) in the DEC-TDI contrast within the SN at the rostral level, in (C) in the boundaries between the dorsomedial and ventrolateral aspect of the SN at the intermediate level, and in (D) in the internal structures of the SN at the caudal level. Anatomical annotations and orientation abbreviation are the same as described in Fig. 2 captions.


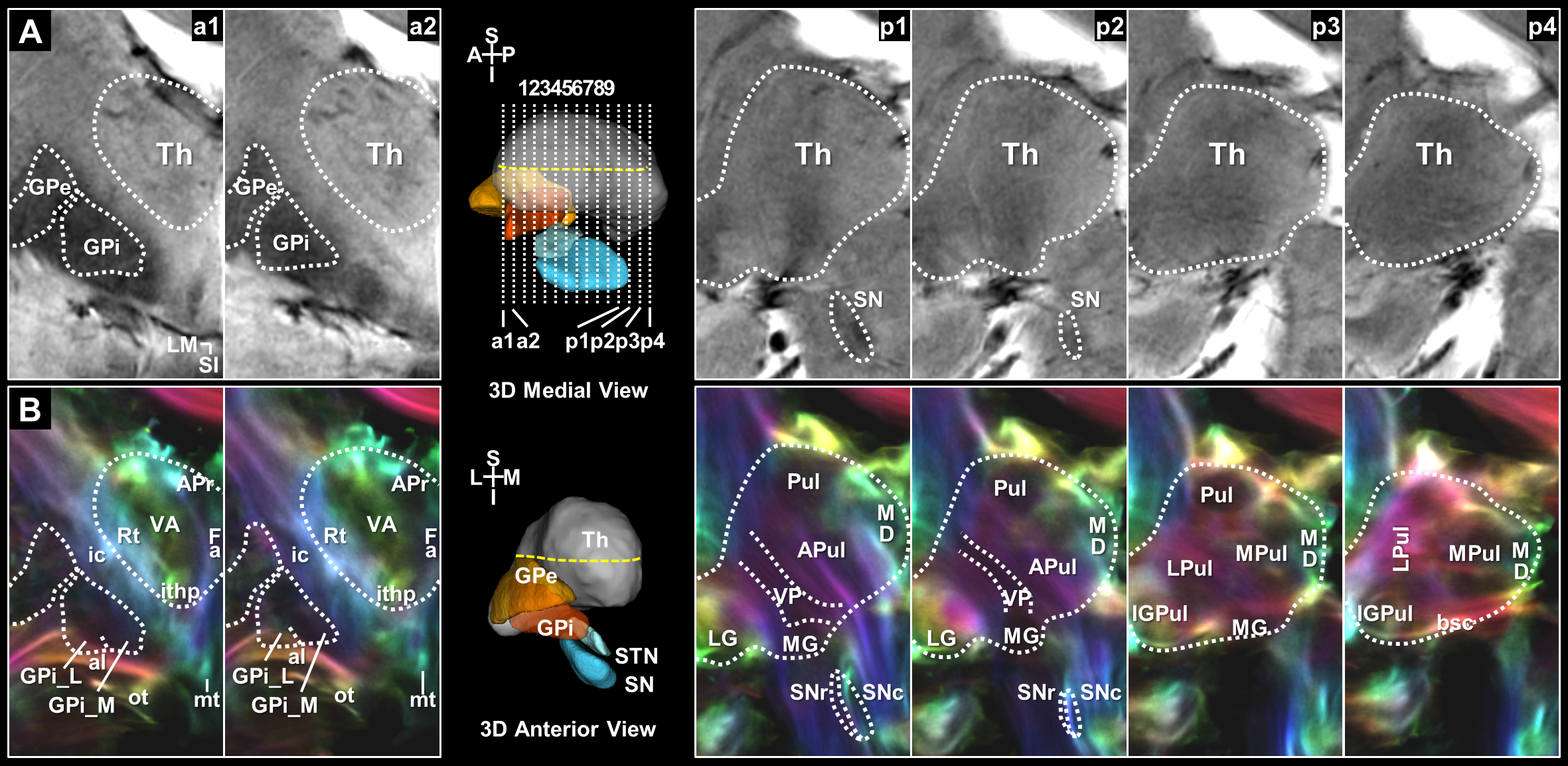


**Supplementary Figure 2.** Additional coronal sections of the deep brain regions and surrounding WM tracts in the super-resolution TDI maps.

(A) The coronal view images of the deep brain on T2*-weighted MRI at 7T in serial 2 mm sections from anterior to posterior levels. (B) Super-resolution DEC-TDI maps with labels of the deep brain regions and surrounding WM structures in selected slices. Anatomical annotations and orientation abbreviation are the same as described in Fig. 2 captions. ithp = inferior thalamic peduncle; MG = medial geniculate nucleus; APul = anterior pulvinar; LPul = lateral pulvinar; IGPul = intergeniculate pulvinar.


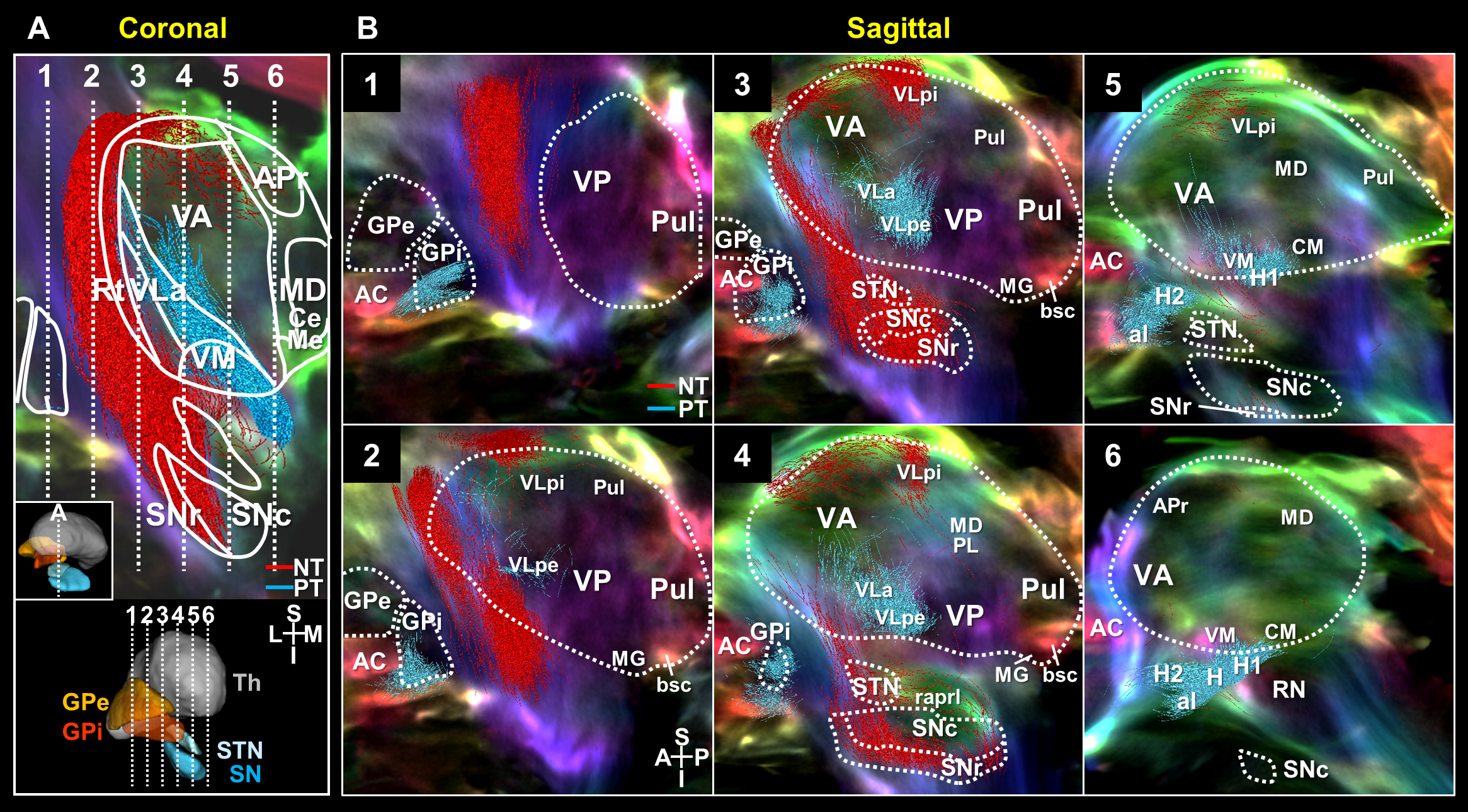


**Supplementary Figure 3.** Sagittal sections of the basal ganglia output pathways.

(A) Sagittal slice levels are shown in a representative coronal section (Fig. 3C-4, top) and a 3D model of the deep brain nuclei viewed from the anterior side (bottom). (B) The DEC-TDI maps are overlaid with the color-labeled pathways (PT: red label; NT: light blue label) reconstructed to 1 mm thickness in serial sagittal views with 3 mm spacing from lateral to medial direction. Anatomical annotations and orientation abbreviation are the same as described in Fig. 2 captions. bsc = brachium of the superior colliculus; MDPL = medial dorsal thalamic nucleus, paralamellar part; MG = medial geniculate nucleus.
